# Free versus subsidised healthcare: options for fee exemptions, access to care for vulnerable groups and effects on the health system in Burkina Faso

**DOI:** 10.1186/s12961-017-0210-z

**Published:** 2017-07-12

**Authors:** Maurice Yaogo

**Affiliations:** 1 Unité de Formation et de Recherche/Lettres et Sciences Humaines, Université Catholique d’Afrique de l’Ouest – Unité Universitaire, Bobo-Dioulasso, Burkina Faso; 2 Association Zama Forum pour la Diffusion des Connaissances et des Expériences novatrices en Afrique (Zama Forum/ADCE – Afrique), Bobo-Dioulasso, Burkina Faso

**Keywords:** Fee exemptions, Access to healthcare, Vulnerable groups, Health system, Burkina Faso

## Abstract

**Background:**

The many forms of healthcare fee exemptions implemented in Burkina Faso since the 2000s have varied between total exemption (free) and cost subsidisation. This article examines both options, their contextual variations and the ways in which they affect access to healthcare for vulnerable people as well as the operation of the health system. This research is part of an interdisciplinary regional program on the elimination of user fees for health services in West Africa (Burkina Faso, Mali and Niger).

**Method:**

A conceptual framework and a chronological review of policy interventions are used as references to summarise the results of the three qualitative studies presented. Historical reference points are used to describe the emergence of healthcare fee exemption policies in Burkina Faso and the events that influenced their adoption. The joint analysis of opinions on options for fee exemption focuses on the different types of repercussions on access to healthcare and the operation of the health system.

**Results:**

In conjunction with the twists and turns of the gradual development of a national health policy and in response to international recommendations, healthcare fee exemptions have evolved since colonisation. The limitations of the changes introduced with cost recovery and the barriers to healthcare access for the poorest people led to the adoption of the current sectorial fee exemptions. The results provide information on the reasons for the changes that have occurred over time. The nuanced perspectives of different categories of people surveyed about fee exemption options show that, beyond the perceived effects on healthcare access and the health system, the issue is one of more equitable governance.

**Conclusions:**

In principle, the fee exemption measures are intended to provide improved healthcare access for vulnerable groups. In practice, the negative effects on the operation of the health system advocate for reforms to harmonise the changes to multifaceted fee exemptions and the actual needs to promote effectiveness and sustainability.

## Background

### Public health policy and healthcare fee exemptions

As a country with limited resources, Burkina Faso faces crucial challenges in the priority sectors for development, namely agriculture, education and health. Public policy often focuses specifically on improving indicators in these priority sectors, and the adoption and implementation of the Millennium Development Goals (MDGs) resulted in several reforms aimed at achieving them. This is the case for the crucial issue of access to healthcare, which has always been difficult to achieve. While direct payment was implemented as the main funding method for the health system in the 1980s in Burkina Faso and other African countries party to the Bamako Initiative, several studies carried out in Africa since the 1990s have demonstrated the negative effects of such policies on the use of services [1, 2], especially for women and the poorest people. Despite an unfavourable context characterised by high poverty rates (40.1% in 2014, down from 46% in 2009 [3]) and indicators that would require significant efforts to be brought up to standards, national decision-makers made it one of their priorities to achieve the MDGs. Meeting such a challenge would require improved and more equitable access to conventional healthcare; as such, a series of decisions [4, 5] since the 2000s have subsidised or eliminated direct payment for certain categories of the population or certain medical conditions.

The structural adjustments introduced by the World Bank and the International Monetary Fund in the 1980s forced African countries experiencing economic crises to reduce their health and education spending. This adjustment to meet international standards had dramatic consequences on the delivery of public services, especially in the field of healthcare. Free healthcare services were supposed to be maintained, but there was a drug shortage in the central pharmacy that supplied drug repositories in regional health centres. New solutions were required, including user-funded healthcare and the introduction of direct payment. In West Africa, this led to the Bamako Initiative, implemented with the involvement of the WHO and UNICEF [6]. The Bamako Initiative brought in direct payment for healthcare in addition to community management and the use of generic drugs. The fees collected were managed by a local management committee consisting of residents of the villages around the healthcare centre, who ordered generic drugs.

In the past 10 years, two options for free or subsidised healthcare have been implemented in Burkina Faso, following the previous actions listed in the chronological review presented below and summarised in the history of the emergence of free healthcare. The first option was the offer of free access to health services, referred to informally as free healthcare in common parlance, which is different from the technical formulation of ‘total fee exemption’ [7]. The second option related to a healthcare subsidy (partial exemption), which varied depending on the disease and the intervention. This subject is examined through a review of three studies as part of a regional research program on the elimination of user fees for healthcare in West Africa from 2009 to 2012. We will not examine a specific case of elimination of user fees for healthcare, as this has been previously performed in the collective work containing the results of this research program [8–11]. In this paper, we will review the similarities and differences between total and partial sectorial healthcare fee exemption (free versus subsidised healthcare) in the context of Burkina Faso. General characteristics common to several countries in this sub-region are discussed by Olivier de Sardan [11], Van Lerberghe and De Brouwere [12]. Our analysis will begin by examining the emergence of a pattern of exemption in the 2000s, which was the starting point for the adoption of several initiatives to reduce healthcare fees. The evolution of official stances on exemptions for various types of care shows opposing viewpoints between the proponents and opponents of a total fee exemption (free) for healthcare. The former defend the merit and necessity of introducing a total fee exemption (free) for healthcare with arguments supporting its feasibility and social utility. The latter advocate a partial exemption (subsidy) as a sustainable and tolerable solution for a low-income country [13]. The results section presents a more detailed description of the sensibilities involved and the arguments developed by both sides.

The research program on the elimination of user fees for healthcare in West Africa was carried out from 2009 to 2012 in Burkina Faso, Mali and Niger. In the context of public healthcare policy reforms in response to the challenges of the MDGs, the simultaneous emergence of fee exemptions appeared as a paradigm shift [14] worth exploring despite the relative lack of research on these issues in West Africa. The reality is that analysis of healthcare policy and systems in the West African context is still in its infancy. This is due mainly to the small number of African researchers, leading to a low scientific output. The Francophone tradition of public health research is also highly medicalised and focused on epidemiological issues, which is not conducive to interdisciplinary questioning around health systems [15].

Initiated as a collaboration among researchers and decision-makers at several institutions in the North and South, the research activities met a need for evidence to inform certain aspects of local policies around access to healthcare [16]. The objective was to gather data on several issues that were neglected or shunned by traditional public health approaches and to fill a gap in the study of healthcare policy and systems. New approaches are needed, especially in health systems governance, stakeholders’ understandings and practices, and the difference between what is stated in official texts and how healthcare services operate in practice. Accordingly, a team coordinated by the author explored various dimensions of public healthcare policy emergence, development, implementation, execution and effects.

The main objective of the research program was to evaluate the removal of direct healthcare payment for certain groups or medical conditions as a policy instrument to promote the use of, and equity of access to, healthcare services. It examined the emergence of public policies to understand the underlying motivations and changes between periods of history. In addition, a review of the different options for fee exemptions adopted over time provided a wealth of information on the effects on access to healthcare for the poorest people and on the health system itself. The conceptual framework and chronological review are used as references to guide the analysis of healthcare fee policy contents.

The first study [13] uses the lens of stakeholders’ and recipients’ understandings to examine the history of free healthcare, focusing on progressive changes in the organisation of the health system. The second and third studies [9, 10] examine the implementation processes of two concurrent policies that increased access to (1) malaria prevention (free insecticide-treated mosquito nets) and treatment (subsidised artemisinin-based combination therapies (ACTs)) and (2) antiretroviral drugs under interventions funded by the Global Fund when compared with national free treatment for tuberculosis (TB).

These studies are reviewed using a related conceptual framework and chronological review to determine the timeline of actions taken as well as the similarities, differences and repercussions of different policy interventions, considering the key themes discussed.

## Methods

Using the conceptual framework, the policy interventions examined in the three studies are compared in keeping with the research program’s methodological guidelines. These guidelines involve a combination of qualitative and quantitative approaches operationalised in multiple-case studies with several levels of analysis corresponding to the national, regional and local levels of the health pyramid and to the types of services by country. The studies referred to here use only the qualitative approach to gather, process and analyse data around the topics studied, using several data sources. Indeed, in addition to documentary data (publications, use of certain public archives), in-depth one-on-one interviews were also conducted with the categories of actors concerned (Table 1).

**Table 1:** Description of the three studies

Type of study	Scale of analysis	Data sources	Interviewee profiles	No. of interviewees
History of free care	National	Scientific literature, administrative archives and print media, in-depth semi-structured interviews	Health, agriculture and education (decision-makers, technicians, implementers)	n = 28
Fee exemptions for malaria (prevention, treatment)	District (Hauts-Bassins region)	Scientific literature, routine data, in-depth semi-structured interviews, group discussions	Decision-makers, local actors (NGOs, associations) and healthcare providers	n = 63
Antiretroviral drug exemptions for children and adults	Regional (Centre and Hauts-Bassins regions)	Scientific literature, routine data, semi-structured interviews	Institutional decision-makers, local actors (NGOs, associations), healthcare providers, service users	n = 30

**Table 2:** Comparative review of the interventions studied

	Form of exemption applied	Government budget commitment	Planning and implementation
Tuberculosis	Free care (except diagnostic tests)	Available budget (Ministry of Health)	- Existence of official texts - Information dissemination via the media and by associations - Institutional communication service
Antiretroviral drugs (ARV) for children	Free ARV without biological follow-up examinations and supportive drugs (varying conditions from one organisation to the next)	No official budget commitment	- No official texts - Limited information dissemination (healthcare centres and associations)
ARV for adults	Free ARV without biological follow-up examinations and supportive drugs (varying conditions from one organisation to the next)	No official budget commitment apart from outside grants (Global Fund)	- Official declaration in late 2009 - Good information dissemination (healthcare centres and associations, media)
Artemisinin-based combination therapies	Subsidised at rates varying by age (2–11 months, 1–5 years, 6–13 years, 14+ years)	Fully funded by Global Fund enabling provision of free care	- Service launched straightaway without prior information (for providers or users) - Application effective as of April 2009 - No official texts
Insecticide-treated bed nets (pregnant women and newborns)	Freely available (distributed to women at pre- and postnatal consultations)	Outside funding (Global Fund) as pilot experiment prior to scale-up	- Unequal conditions for information dissemination; limited to women attending postnatal care services
Insecticide-treated bed nets (2010 national distribution campaign)	Freely available (quota for all households counted)	Fully funded by Global Fund (Programme d'appui au développement sanitaire and Plan Burkina)	- Contractual obligations (Global Fund funding requests) - Communication campaign in the public media (radio, TV, print media) - Existence of a timetable for directives and conditions for implementation but targets not met

The purpose was to understand how the elimination of user fees was implemented, its strengths and weaknesses, the perceptions of different categories of stakeholders including healthcare providers and patients, as well as the strategies of social actors. To do so, data was gathered and analysed in detail to obtain a better understanding of the point of view of social and professional stakeholders and to collect common views of the topics discussed [17]. The data collected concern both the current perceptions and practices of political and healthcare actors in relation to interventions and public policy on healthcare fee exemptions.

**Fig. 1:**
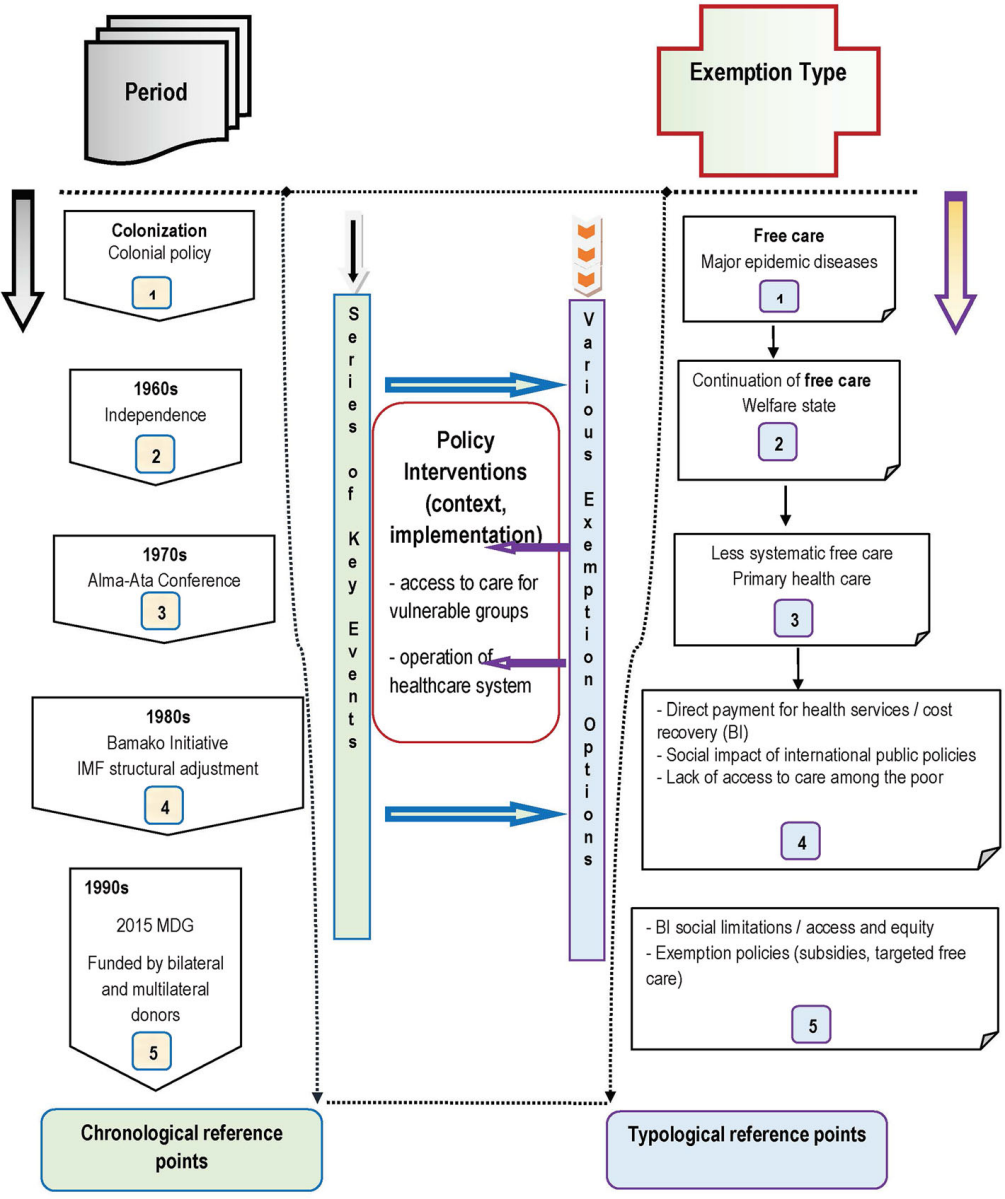
Timeline of healthcare fee exemptions in Burkina Faso. Chronology of the evolution of healthcare fee exemption policies in Burkina Faso

**Fig. 2:**
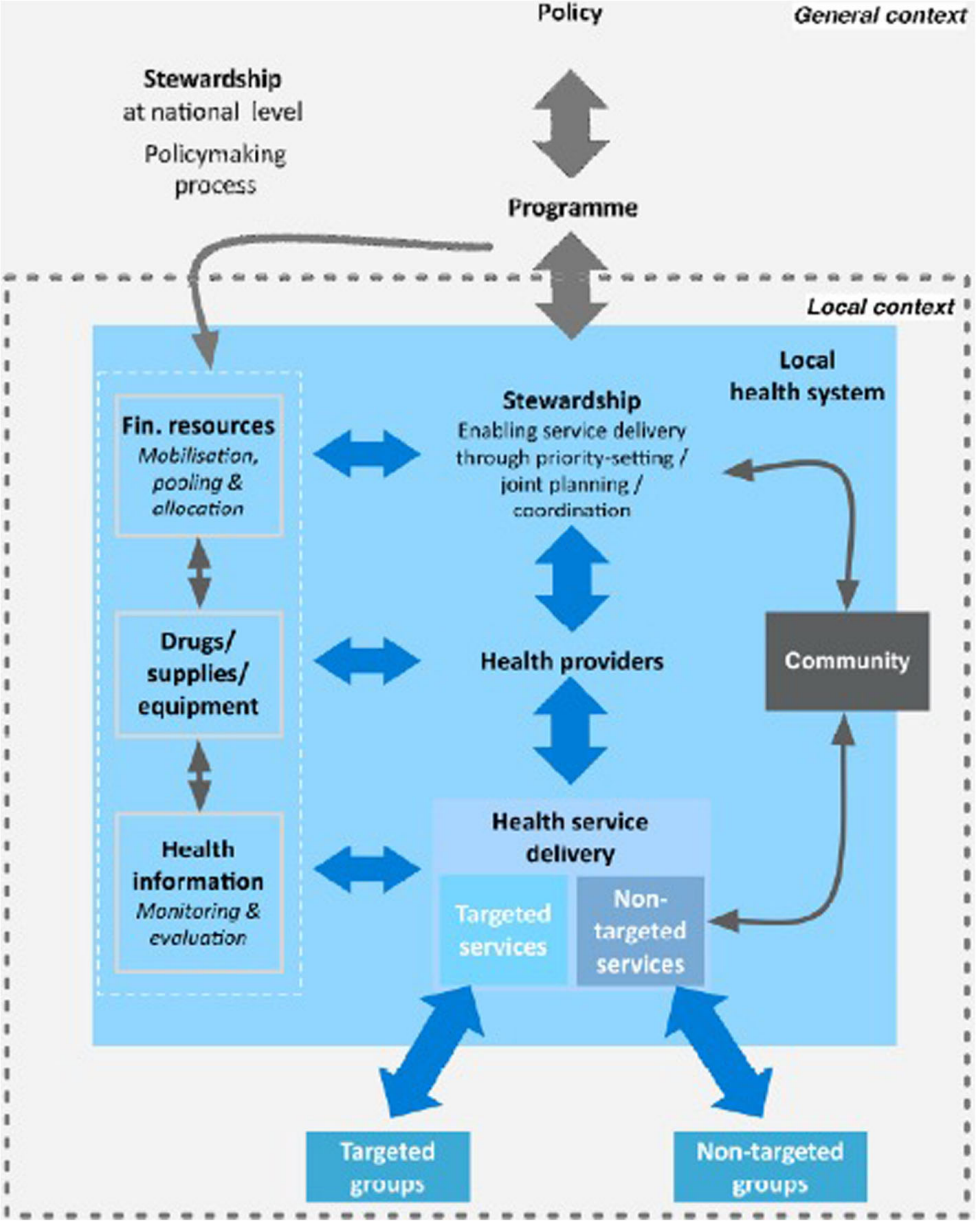
Conceptual framework for the analysis of the effects of a specific policy on the local healthcare system. Source: Van der Veken et al. [18]

Figure 1 shows the timeline of fee exemption options. The chronological review of policy interventions describes the succession of events that have affected healthcare fee exemption options since medicine was introduced during the colonial period. It provides a historical anchoring for successive fee exemption options, showing the events related to colonial exploitation policies, followed by the implementation of national health and development policies, and provides the basis for the conceptual framework (Fig. 2). In effect, it lists the real facts that paved the path to the current options of eliminating or subsidising healthcare fees.

The description of the nature and content of exemption options based on typological reference points shows the progressive changes in relation to the historical events that affected the emergence and formalisation of national health policy. Here, the main idea is to provide the information that best describes the configuration of total and partial healthcare fee exemption policies in reference periods connected to the chronological axis. The historical reference points are thus associated with the successive options, reflecting the typology of fee exemption policies and their structural (e.g. change from total fee exemption to cost recovery) and economic changes (e.g. recent measures in response to the need of vulnerable groups for more equitable access to healthcare). This highlights the implications on several levels. Our interest here is in access to healthcare for vulnerable groups and the effect on the operation of the health system.

The proposed chronological review can be applied to the analysis of other sectors in that it provides a dynamic link between evolving historical facts and their impact on the formalisation of public policy.

The conceptual framework is that developed and used by Van der Veken et al. [18] to analyse the effects of a healthcare fee exemption policy on the local (district) health system. The main idea is that the six essential functions of a health system defined by the WHO [19] do not sufficiently account for the dynamic interactions between the various components. The proposed conceptual framework describes the building blocks of the local healthcare system and their interrelations from a different perspective.

The central axis of the model proposed by Van der Veken et al. [18] for the analysis of the fee exemption policy on the local health system (policy effects mapping) consists of four key elements, namely governance/stewardship, personnel responsible for healthcare provision, healthcare delivery and the community. By contrast, drugs, supplies, financial resources and the health information system are considered resources of the central axis. All components are interrelated, and the effects of a policy targeting one particular component can have (expected or unexpected, positive or negative) effects on another component, which reflects the system’s complexity [20].

Based on this model, which complements the chronological review presented above, the interest is in the impact of sectorial fee exemptions for the service offer (the official package) on target groups, specifically the effect on the barriers to access for disadvantaged groups and the positive and negative consequences for the health system. Another important dimension is the source of financial resources (international donors, national institutions), which affects the duration of sectorial fee exemptions.

## Results

A general summary of the results of all three studies is presented, referring us to the two thematic axes (the contextual and typological reference points) depicted in the chronological review. First, we give a broad overview of the key events that influenced decisions in favour of different forms of fee exemptions. Second, we examine the context of the emergence and formulation of these options for the elimination or reduction of service fees, highlighting their similarities and differences. Third, we discuss the various perspectives of different socio-professional categories, examining the issues with these exemption measures in terms of the projections made for meeting health objectives, which is the focus of this article.

### Public policy and healthcare fee exemptions: some chronological reference points (first study)

As the proposed chronological review shows, there are five main periods in the historical evolution of free healthcare:

- The colonial period, when free healthcare was provided jointly by the colonial administration and Christian missions (1920–1960);
- The Independence period, when healthcare continued to be free and the health issue was integrated in the implementation of specific development plans;
- The international healthcare reform period (health, economy) with the introduction of structural adjustment policies that negatively affected healthcare and other social sectors, and cost recovery under the Bamako Initiative;
- The questioning period, embodied by international awareness of inequality and inequity, as well as large-scale initiatives focused on improving access to healthcare (MDG, Global Fund, etc.);
- The current period, which began in the 2000s with the introduction of several sectorial healthcare fee exemptions.

This section outlines the different types of healthcare fee exemptions implemented in the abovementioned reference periods. To make the connection to the chronology of public policies adopted, the following important dates can be used as references:

1. The implementation of the *Plan cadre* (Development Policy Framework Plan; initially from 1967 to 1970, then from 1972 to 1976) for sectors considered as priorities for local development (agriculture and the economy) as opposed to so-called social sectors (health and education);
2. The adoption of the first *Code de santé publique* (Public Health Code) in 1970, updated in 1994 in the form of a law passed by the National Assembly to specify the regulatory provisions and conditions of practice for different human health professionals;
3. The implementation in 1978 of the first *Document de programmation sanitaire nationale* (National Health Development Document) with the support of WHO, which would serve as the basis for developing health policy [21];
4. The development of the *Document de politique sanitaire nationale* (National Health Policy Document) [22];
5. The development of the *Plan National de Développement Sanitaire* (National Health Development Plan) from 2001 to 2010, followed by an extension from 2011 to 2020;
6. The official adoption of the first *Politique nationale de santé* (National Health Policy) [23].

While it is difficult to make a direct connection between the introduction of health policies and fee exemption measures, a break with previous practice occurred with the introduction of the subsidy policy for deliveries and emergency obstetric and neonatal care in 2006. In effect, the long-term financial commitment to action at the national level was adopted in the country’s budget under the 2015 MDGs. However, there were other significant developments before this one. One of the reference documents [24] gives an overview of previous experiments, including a subsidy for emergency obstetric care in hospitals and the cost-sharing initiative.

Various forms of policy interventions in the field of healthcare have been implemented since the introduction of modern medicine in the 1930s. To understand the developments up to the present day, a brief overview is useful to get a sense of the twists and turns of the progressive implementation of health policy. This helps to highlight the main milestones in healthcare fee exemption since the time when the government was being formed after colonisation. Up to independence, health activities were part of a “*broader perspective of socio-economic development in general*” (translation [21], p. 2; [25]). The first development plan, the *Plan cadre*, was realised in the period from 1967 to 1970, after several unsuccessful attempts from 1962 to 1966. Several publications have demonstrated a link between two sectors in this plan, those of health and agriculture; hence, the large-scale health interventions (fight against endemic diseases) preceded agricultural development in this period. At the time, the control of endemic disease and the development of an agricultural framework were identified as agricultural policy targets, with the goal of training agricultural entrepreneurs to farm irrigated areas developed across the country. Similarly, large areas of fertile ground were infested with onchocerciasis and could not be cultivated until after widespread action to fight major endemic diseases [26].

The outlines of a general health policy and a personnel policy were defined in the *Plan cadre*. The first direction mentioned both the pursuit of programs to control certain communicable diseases and the strengthening of basic public health activities (statistics, basic education, nutrition and sanitation). The second direction was on bolstering training for personnel, the required investments and the funding sources. The recommendations from the first *Plan cadre* were repeated with only minor changes for the second, which covered the period from 1972 to 1976. They were used as a reference to develop the first *Document de programmation sanitaire nationale* for the period from 1980 to 1990. Up to the end of the 1980s, the Ministry of Health’s actions were guided by the 1970 *Code de santé publique*. This code was limited to regulating medical services and care. The March 1979 adoption of the recommendations of the Alma-Ata Conference on primary healthcare is considered the starting point for true healthcare policy, with the subsequent development of the *Document de programmation sanitaire nationale*. In line with the overall direction, which was to provide better health coverage to the population, a pyramidal healthcare structure was planned, which is still used as a reference today: (1) one primary care facility (PCF) per village, (2) one centre for health and social advancement (CHSA) per 15,000 to 20,000 people in a maximum 20-km radius, (3) one medical centre per sub-prefecture, (4) two national hospitals in the two largest cities (Ouagadougou and Bobo-Dioulasso), and (5) one school of health sciences. Currently, the first level of the health system is not the PCFs, which are no longer in operation, but the CHSAs. There has also been a change in the spatial distribution of health structures due to changing administrative boundaries and the subsequent reorganisation of the health system, as well as the adoption of an agreement to implement health districts as functional units in the health system. Education of medical personnel takes place mainly at two public universities, initially Ouagadougou and then Bobo-Dioulasso, and more recently in private institutes certified by the government and the *Conseil Africain et Malgache pour l’Enseignement Supérieur*. Three public health research institutions were also created, including the Muraz Centre in Bobo-Dioulasso in the 1940s, to fight major endemic diseases.

The main aspect regarding changes in the organisation and operation of the health system is that, after the notorious centralisation of 1960 to 1979, a progressive decentralisation movement began. There were four different periods in these developments [27]:

1. The period from 1960 to 1979, which was the centralisation of health administration.
2. The period from 1980 to 1989, which was the progressive decentralisation of the health administration and the beginning of technical decentralisation. This was when legislation began to grant autonomy to public health services, for example, as industrial or commercial public institutions or semi-public companies. In the pharmaceutical sector, this was the case with the *Office national d’approvisionnement pharmaceutique*, which became a government corporation under the name *Société nationale d’approvisionnement pharmaceutique* in 1985 and was then privatised in 1994.
3. The period from 1990 to 1993, which is described as a time of technical decentralisation, with an increase in the number of districts from 45 to 53; today, there are 63 districts.
4. The current period, which is considered a time of increasing health decentralisation with challenges. After the health system was organised into districts by a November 30, 1993, departmental order, changes were introduced in 2001, dividing the country into 13 administrative regions used as a reference to reorganise the health system. The opportunity to combine territorial and health services decentralisation led to a debate on the functional efficiency of this option.

In the context of progressive changes in the organisation and operation of the health system, the fee exemption policies, as they are applied today, are described in reference to the development of two major policy interventions. These policy interventions had a significant effect on the decisions to subsidise or eliminate healthcare fees. The first major intervention consisted of the progressively formalised actions to provide a foundation for the key aspects of health policy based on the directions from the Alma-Ata Conference on primary healthcare (in 1987) and the recommendations from the Bamako Initiative (in 1987). These two initiatives with converging objectives were used as models during the post-colonial period until the introduction of sectorial healthcare fee exemptions. Accordingly, we will describe the introduction of primary healthcare as following a paradigm of community participation and direct payment for healthcare at the point of service. This paradigm precedes that of the healthcare fee exemption, which came about through multiple policy interventions since 2000. A few challenges in applying these policies will be reviewed to show what led to a paradigm shift dictated by the barriers to healthcare access, especially for the poorest people.

According to WHO [19], “*Primary health care is essential health care based on practical, scientifically sound, and socially acceptable methods and technology made universally accessible to individuals and families in the community through their full participation and at a cost that the community and country can afford to maintain at every stage of their development in the spirit of self-reliance and self-determination. It forms an integral part of the country’s health system, of which it is the central function and focus, and of the overall social and economic development of the community*.”

The Bamako Initiative was an approach to strengthen primary care through what is known as community participation in funding and managing healthcare services. The sale of essential generic drugs was the main path to primary care funding. The underlying mechanism in the Bamako Initiative was cost recovery through the involvement of local users. To start, an initial repository of drugs was provided to the population, and local management structures were asked to sell essential generic drugs to users at affordable prices with a modest profit margin. The profits were used first to replenish the repository from the distribution structure, which was the *Centrale d’achat des médicaments essentiels génériques* (CAMEG). The rest was used to fund certain costs in health structures and provide bonuses to healthcare personnel. For the Bamako Initiative to be successful, significant involvement from the population was required. Stakeholders needed to be trained to define healthcare activities and manage finances. The main objectives were to make services more efficient, limit costs and provide continuity of care while the health system was reorganised.

Concerning the adoption of primary healthcare in Burkina Faso and several sub-Saharan African countries, experience shows that a solution was proposed to resolve healthcare problems in developing countries, specifically in sub-Saharan Africa. This simultaneously signalled a questioning of previous initiatives and their limitations and failures, particularly in terms of prevention and mass medicine (in the case of the high prevalence of communicable diseases and high maternal and infant mortality) and the low efficiency, high cost and dysfunctionality of hospital medicine [28, 29]. However, the idea of primary healthcare was much criticised in that its initial form suffered from ambiguity and lack of precision, especially in the expressions ‘scientifically sound’, ‘socially acceptable’, ‘universally accessible’, ‘community’, and ‘cost that the community and country can afford’. The possibility of adapting medical practices to local conditions was raised, specifically for low-income countries. However, a highly optimistic culturalist reading was made of the ways in which local stakeholders would be involved, based on local, collective or community consensus around health decisions and actions [30, 31]. One of the reasons why primary healthcare was initially rejected in Burkina Faso is related to its politicisation by the progressive regime from 1983 to 1987, according to a populist strategy by the central government to mobilise farmers by delegitimising the power of traditional chieftainships [32], leading to conflict between government structures and traditional leaders. Thus, the difficulties of implementing a primary healthcare approach in Burkina Faso using PCFs were two-fold – on the one hand, social issues stemming from confrontations between the central government and traditional chieftainships, and on the other, the poor conditions of service that failed to satisfy users, leading to low use of PCFs. This cumulated in the abandonment of this strategy and a reorganisation of the structure of the health system with CHSAs replacing PCFs as the first level of the structure.

In both the Alma-Ata and Bamako recommendations, the idea of local medicine came through in the proposals, but planned actions, as formulated in the recommendations, were highly influenced by the prevailing ideology of community participation. In principle, the objective was for local healthcare structures to be operational and self-managed by involving the population in their operation to better meet health needs. In other words, the issue was how well the local population could meet its health needs. The initial procedure when medicine was introduced to the country was to provide certain services, notably by being cost free, during colonisation and the first few years of independence. After this period, it became necessary for local users to be responsible for their own health needs, which led to the idea of ‘participation’, with its ideological connotations and biased interpretations.

The Bamako Initiative was implemented in public structures using several different cost recovery methods, including drug sales, user fees at the time of service, the implementation of medical plans, and the creation of prepayment and mutual health insurance systems. These systems all have two things in common – on the one hand, their goal is to raise financial resources for public healthcare structures facing significant restrictions in the government’s budgetary envelope, and on the other, they fail to consider the inability of most rural households to pay for healthcare based on the fee structure applied [33]. In practice, the fee for public hospitals is a deterrent for a large portion of the population. The lack of operational mechanisms for providing care to those in need is another barrier to healthcare access for the poorest people, who are often excluded from access to conventional healthcare. Cost recovery was an important step in the development of health systems and the delivery of care. The new organisation introduced to ensure community involvement through management committees was the backbone of the new mechanisms, based essentially on charging fees for care and selling less costly generic drugs from decentralised repositories. The Bamako Initiative implementation conditions, results and issues that led to a gradual return to sectorial or adjusted fee exemptions are well documented in the literature [34, 35].

It is generally agreed that the Bamako Initiative was not a bad policy, but it was implemented in an increasingly adverse context due to the decay of both public finances and healthcare structures, with a dramatic drop in the quality of care. The social repercussions were therefore already in sight, especially the exclusion of the poorest people from care due to the direct payment system. Without a mutual or other health insurance system, the poorest people, already struggling to survive, were clearly unable to pay the required fees for the healthcare services on offer. The issue of access to healthcare remains separate from the involvement of management committee members, which has shown certain functional benefits for healthcare services.

In principle, free healthcare delivery worked in a fashion similar to the time of colonial administration and Christian missions in Africa. These were all cases of free healthcare funded by outside subsidies or allocations, whether by direct investment (donations from governments and international institutions) or charitable works (many forms of donations and aid) through the Christian missions. The operating mechanisms were also similar, since the goal was to reach users in various ways without any direct payment. On this basis, healthcare was gradually implemented throughout the territory starting with these two levels of intervention.

When new countries achieved independence in the 1960s, free care operated according to the same initial principles, namely free healthcare funded entirely by public resources, with additional support from outside donors. Drugs were supplied from a central repository, which became the CAMEG. The free healthcare that applied during this period included several levels of access to services and of involvement of healthcare providers. In effect, access to care was free or mandatory, delivered by a permanent or mobile clinic, for specific needs (epidemics and other common diseases), and provided by the government network or through the Christian missions. Patients paid no fees for treatment, including being placed under observation or hospitalised if needed, vaccinations for prevention, and access to drugs. The initial healthcare fee exemption led to a ‘welfare state’ philosophy that is remembered to this day.

During the time of primary healthcare and the Bamako Initiative in the 1980s to 1990s, the implementation of a new functional organisation of health centres (health centre management committees, drug repositories and a more significant role for local stakeholders) marked the beginning of healthcare privatisation. The government no longer directly intervened, except to fund infrastructure and wages and to train healthcare professionals and develop basic documents. The government’s disengagement was dictated in part by international structural adjustment policies that insisted on less funding for the social sector relative to the economic sectors. These choices preceded the current situation, with barriers to healthcare access for the poorer strata of society. In such a context, a return to fee exemption policies of various types is readily explicable.

The current stage in the operation of the health services is a return to free care sparked by globalisation and the internationalisation of health decisions and actions [36] and made possible by large-scale mobilisation of external resources. This was made possible by the support of foreign aid (the European Development Fund, the Heavily Indebted Poor Countries Initiative, the Global Fund, etc.) and of initiatives related to the MDGs. These external health funding initiatives ask the still unresolved question of who is responsible for local public policy, given the need to maintain financial commitments for long enough to have lasting effects. Funding for the fight against HIV/AIDS is an eloquent example of how structures that become familiar in the institutional landscape are directly connected at the highest level of government (presidency). They therefore operate with massive resources from international donors (in particular, the Global Fund) that are too large to be used and controlled by the Ministry of Health’s usual decision-makers. This upsets the balance of operations in this department, with many relatively autonomous vertical programs that are difficult to coordinate and synchronise with sectorial strategies.

A further area where free healthcare is applicable in Burkina Faso examined in our chronological review concerns the major endemic diseases, specifically onchocerciasis, trypanosomiasis, leprosy and TB. Other than the description of the unequal contribution of colonial medicine to treating these recurrent diseases in the past [37], testimonies highlight the commitment of stakeholders in this period of great health challenges and of multiple interventions [38]. Testimonies also described the practices used during the epidemics [39] that have wreaked havoc, some of which continue today (meningitis, measles, yellow fever). The importance of significant actions in the process of introducing medicine were an argument in favour of biomedical care, despite the initial resistance, which sometimes led to the need to use skills (e.g. mobile teams) or force, depending on the circumstances.

The fight against the major endemic diseases was the starting point for health action at a time when they were cancelling out the efforts of colonial development. Trypanosomiasis, TB, leprosy and onchocerciasis were all formidable foes that truly threatened both the population, which were constantly exposed, and the local production objectives involving this population, leading to the efforts used to eradicate them. The colonial policy response was appreciated for its effectiveness, which set the foundation for the reputation of modern medicine as having the means to stop the spread of these devastating epidemics. The same applied to preventive care for some of these diseases, which was delivered through mass vaccination. However, the main purpose of these campaigns was to stop diseases with a high potential to become epidemic, contrary to other conditions like malaria, which persists today.

The example of the program to fight onchocerciasis illustrates the important contribution of interventions initiated to fight major endemic diseases. This was indisputably one of the largest health projects carried out in Africa. Launched in 1974 to eradicate the disease, initially in an area covering seven countries (Benin (previously Dahomey), Burkina Faso (previously Upper Volta), Ghana, Mali, Niger and Togo), the program was later extended to four other countries (Guinea, Guinea-Bissau, Senegal and Sierra Leone). More than 30 years after it began, it is considered one of the most effective regional health programs ever implemented. This large-scale health intervention was sponsored by the major development institutions, including The Food and Agriculture Organization of the United Nations, the United Nations Development Programme, WHO and the World Bank. They were involved to pursue two goals, both health-based and economic, namely to eradicate this communicable disease and to promote the economic development of areas freed from the disease. The health goal was met, since onchocerciasis is no longer a threat to public health in the initial intervention countries.

The previous reality was that many villages were abandoned due to the high prevalence of the disease in the most exposed areas, namely areas near rivers and streams with significant onchocerciasis infestation. However, the results of several studies [40] show that villages were abandoned for reasons other than onchocerciasis outbreaks. A distinction must be made between the fact that populations also move from areas not affected by onchocerciasis, demonstrating regional demographic change for reasons not related to health, and the fact that, in residential areas with onchocerciasis, prevalence of the disease was certainly one of the causes for abandoning so-call ‘front-line’ villages.

After healthcare fee exemptions ended during the colonial and postcolonial period (up to the 1970s), access to healthcare remained free under vertical programs for a few target diseases, including TB, leprosy and onchocerciasis. Although these major endemic diseases were eradicated on a large scale, they were still present at a lower prevalence than is considered in health policy. This was also the case for mass vaccinations against epidemics and vaccines for women of childbearing age and infants. There were also no fees for pre- and postnatal exams, preventive malaria care and the treatment of severe malaria. The wave of partial fee exemptions in the 2000s completes this panorama of actions undertaken to improve users’ access to healthcare. For many sub-Saharan African countries, the 2000s were a clear turning point in terms of the implementation of healthcare fee exemption policies for vulnerable sectors of the population. In response to the limitations and failure of cost recovery as recommended by the Bamako Initiative [6] and the Alma-Ata Conference on primary healthcare, a cascade of exemption initiatives was introduced. In Burkina Faso, these initiatives initially targeted pregnant women and children under five, and some were later extended to other categories of users.

A comparison of the different data sources listed shows that economic concerns are what led to new interventions based on sectorial fee exemptions to avoid excluding the poorest people from access to primary healthcare. The subject of this article emerged from the opinions expressed about the adoption of policies that favour one side of the healthcare fee exemption pole – free or subsidised. Accordingly, the issue is addressed by putting into perspective the different stakeholders’ (policymakers and health professionals) contrasting points of view in favour of one or the other of these options, not the views and opinions of the communities served, which naturally prefer a total fee exemption for healthcare. The following section gives more information on the emergence and formulation of fee exemptions for the interventions discussed in the two other studies.

### Emergence and development of sectorial exemptions for malaria and HIV/AIDS

#### Malaria (second study)

Like for other services and diseases, several partial and total fee exemption measures for malaria care have been adopted in Burkina Faso in the past 12 years. For example, ACTs have been subsidised since 2006, and long-lasting insecticide-treated nets (LLINs) are distributed for free.

From a chronological perspective, there were other fee exemption policies for malaria care before the current ones came into effect. First, two centres for malaria research and intervention were created, the first in Bobo-Dioulasso (the second-largest city in Burkina Faso) in 1941 and then in Ouagadougou in 1987. Then, the country subscribed to several international recommendations, including those of the 2000 African Union conference in Abuja and the 2002 Roll Back Malaria Initiative, leading to the implementation of specific action plans and programs. More recently, Burkina Faso’s requests for the malaria component in rounds seven (2007) and eight (2008) of the Global Fund to Fight AIDS, Tuberculosis and Malaria allowed it to acquire significant funding for several forms of service fee exemptions (48 million USD from 2003 to 2008 [41]). The strong commitment of this organisation and other cooperation institutions allowed Burkina Faso to introduce free healthcare for certain services.

The decision to subsidise the cost of ACTs was made when chloroquine was replaced in 2003 following WHO guidelines. However, the initial local sale price of 3000 FCFA (6 USD) was deemed high, and less costly generic drugs were not available. In 2006, the World Bank began providing funding to subsidise the treatment (at 100 FCFA or 0.2 USD) for children under five, at a time when free treatment for severe malaria had already been adopted. Later, extension of the subsidy to adults became possible due to the resources acquired in round eight of the Global Fund, which allowed the scaling up, with a community component, of the distribution by community health officers of LLINs and subsidised drugs. The agreement between the World Bank and the national institution in charge of managing the common basket of external funding (*Programme d'appui au développement sanitaire*) to implement policy interventions before rounds seven and eight of the Global Fund stated that cost recovery would be used only to fund the resupply of inputs and not for other needs such as equipment or personnel management.

One of the major initial challenges was the stock-up of sufficient drugs to meet the needs of both those initially covered (pregnant women and children under five) and those who would be covered once the policy was scaled up to the entire population. The initial stock for the first component of the intervention, through World Bank funding for pregnant women and children under five and Global Fund funding to subsidise sale to the community, was successfully gathered. However, ACT supply difficulties plagued the scaling up starting in 2008 because there were not enough ACTs on the international market and distribution based on the common basket principle did not respect the availability per source of funding.

During the same period, two preventive policy interventions to distribute free LLINs were implemented. The first made LLINs available to health districts for pregnant women who came in for pre-natal exams following a 2002 official decision introducing free preventive healthcare for vulnerable groups. Starting in 2008, grants from the Global Fund and other cooperation organisations, including the Canadian Red Cross, made it possible to extend the policy to children under five. The second policy intervention for free distribution of LLINs occurred across the country in 2010 and was made possible due to funding acquired in round eight of the Global Fund (26 billion FCFA) to scale up the intervention. The involvement of a non-governmental organisation (NGO) (Plan Burkina, now Plan International) with regional actors (four national NGOs) independent from the health system was a new experience of private–public partnership.

The sudden implementation of tailored exemptions, without sufficient preparation for stakeholders, occurred without the appropriate formal provisions, in contrast with the existing mechanisms for other interventions (e.g. free TB treatment). Reference texts were also produced during the preparation and formulation process for the maternal healthcare subsidy decided on in 2006.

#### Sectorial exemptions for TB and HIV/AIDS (third study)

##### TB

Since the 1950s, treatment for certain endemic diseases like TB has been free. Later, in the 1980s, political and social issues related to the HIV pandemic gradually forced countries to introduce free antiretroviral drugs (ARV). TB treatment had been free since the colonial period as part of the large-scale fight against major endemic diseases [42]. Like for other diseases, TB treatment continued to be free after independence (1960) to promote access to treatment, but the incidence of TB increased with the appearance of HIV.

For AIDS, free ARVs were implemented in Burkina Faso in 2009, much later than in other countries like Senegal (2003) and Mali (2004). Financial access to treatment had been a problem since the first drugs became available in 1996. It is recognised that activism by associations promoting access to drugs in the global South is what considerably brought down the prices of ARV, and later made them free in most sub-Saharan African countries.

There were two main phases to free TB treatment that demonstrate the evolution of public policy in this area. The first phase occurred during the colonial period, and the second, which is ongoing, is based on WHO recommendations under the coordination of the *Programme national de lutte contre la tuberculose* (PNT). Other than the description of the unequal contribution of colonial medicine to treating these recurrent diseases in the past [37], testimonies highlight the commitment of stakeholders in this period of great health challenges and of multiple interventions [38]. Later, starting with independence in 1960, treatment was provided by TB treatment centres, with large centres in the two largest cities and five smaller centres in secondary cities (Koudougou, Ouahigouya, Fada N’Gourma, Dédougou and Banfora). Since treatment was highly centralised, people with TB often had to travel great distances for diagnosis and treatment, at a time when treatment was not yet standardised. During this time, free care meant hospitalisation, laboratory tests and X-rays during treatment and monitoring, as stated in the main official documents. However, experience shows that the implementation of exemption measures was dysfunctional due to input shortages and no budget line reserved for TB treatment. As a result, patients had to pay, but the amount differed depending on the structure. In addition to drugs, certain tests, including X-rays of the lungs, were paid, meaning that provisions and practice did not match in real time.

Before the PNT was created in 1995, another step occurred, with better organisation of care and effective application of free care. With support from WHO, other partners and the government, who subsidised drugs, the PNT had significant resources to implement its guidelines. Its role was to coordinate the activities in different diagnostic and treatment structures at different levels of the national health system (regional health branches, health districts, TB diagnostic and treatment centres). It was supported by reference centres for the treatment of drug-resistant TB, the *Programme d’appui au monde associatif et communautaire*, traditional medicine associations and former patients.

##### HIV/AIDS

The payment exemption for HIV/AIDS treatment was initially provided for children through the NGO ESTHER, starting with a 2003 pilot program to provide medical care for 200 children in three medical centres in Ouagadougou (n = 91), Bobo-Dioulasso (n = 30) and Ouahigouya (n = 17). In 2008, gradual decentralisation of medical care began in certain regional hospitals, district hospitals (medical centres with surgical units) and association-led medical centres. ARVs were supplied by the *Comité interministériel de lutte contre le sida*. Associations also provided aid to parents with infant formula, the purchase of drugs to treat opportunistic infections and payment of biological testing fees. All healthcare providers indicated that ARVs were free [43], but hospitalisation, biological testing and drugs to treat opportunistic infections were not; these costs differed from structure to structure.

Free ARVs for adults were introduced in late 2009 in response to significant pressure from many AIDS organisations organised in very active networks. They were assisted by other converging actions, including Amnesty International’s 2009 plea for free maternal healthcare [44].

### Free versus subsidised: a comparison of actors’ and users’ arguments

The perceptions of different forms of healthcare fee exemptions are not unrelated to common opinions on free things in general. The same goes for perceptions elicited by official guidelines. As the chronological reference points listed above show, free healthcare was introduced during colonisation and modified after independence. The connotations of the word ‘free’ in one of the three main languages used, as reported by an informant, illustrate the negative perceptions attached to it. In Mòoré language it is said that the word *zaalem* connotes “*zero, nothing, gift*” or “*no reason*” [45], reflecting certain traditional cultural values. As another informant said, [translation] “*We say that when something is free it’s not good, and when you pay it’s good*” (Primary school teacher). Based on these markers of the influence of local traditions, individuals attempt to care for their own health and that of their loved ones, or at least contribute to preserve their human dignity. The same applies to basic needs. Certain local traditions still have an influence, though given the constant changes brought about by increasing modernity and the multifaceted types of development, they do not dictate the population’s behaviour [46].

Reactions are comparable in some respects, especially the frequent criticisms of free healthcare over subsidies that include a small contribution from users. Just like health system actors who have similar reactions, the delivery of free healthcare without a symbolic contribution from recipients is questioned, sometimes because of cultural significance and other times because the recipients do not believe the system would be sustainable in the long term. Similarly, an informant made the following comment about an agricultural initiative: [translation] “*Even if it’s 10 francs, even if it’s 25 francs, it needs to be used to educate, to give meaning and value to seedlings; even reforestation costs something. It costs something, it has its price; even if it’s subsidised, we should not develop the idea that everything is free*” (Former policymaker and head of a public institution).

Both healthcare providers and certain informants also testified that free care was poorly used because of the notion that anything offered for free is of less value. Furthermore, some behaviours described went against expectations or assigned objectives, including abusive use of healthcare services or, alternately, distrust or clear reluctance, failure to use drugs not seen as legitimate treatment (generic drugs are a known example), and waste of subsidised agricultural inputs (seed given as feed to animals is a clear example). Although this type of marginal behaviour is observed, it is relative, given the positive effects on most users, who take prescriptions as directed, and the broader benefits noted by proponents of free healthcare.

A similar reasoning states that free healthcare is incompatible with a long-term solution or that users are less ‘accountable’ when healthcare is free [13]. Similar opinions are expressed by stakeholders disappointed by misuse of free healthcare or its adverse, difficult-to-control effects (implementation without adequate support, input shortages, personnel and equipment shortages, supposed threat to the solvability of public resources). On the other hand, the informed but less numerous proponents of selective fee exemptions for certain services defend the merit of equitable access to healthcare achieved by providing free or lower cost healthcare to disadvantaged populations, but not to other user categories. As for the users, some are nostalgic for the welfare state [47], and many expect at least reduced healthcare costs, if not free healthcare. These results show that healthcare providers and other categories of informants have different perceptions of free healthcare. One side says it gives the poorest people access to healthcare; the other side says free care is misunderstood and devalued by users, leading them to conclude that subsidised care is better, with its minimal user contribution.

### Certain informants who rejected free healthcare envisaged other options, such as:

1. A sliding scale approach, where fees are determined by users’ ability to pay and the government’s limited capacities, while maintaining a satisfactory quality of care, as illustrated by the following comments:
  [translation] “*There are some people who really have nothing … for them, it should be free. But I don’t think that everyone should expect it to be free. At some point, the government can’t support it. I would also say, by saying that I’m against free healthcare, it’s because I want health services to be able to improve both their delivery and structures*” (Teacher and head of a civil society organisation).
2. Another informant proposes a temporary and sectorial disease-based approach focusing on diseases requiring urgent action:
  [translation] “*I think we could further reduce costs and try to combat the deadliest diseases like malaria and HIV. These could be 100% covered until the disease is really under control or eradicated, until we can return to normal prices*” (Head of a national health program providing fee exemptions).
3. A formula where drugs are subsidised with social protection mechanisms is suggested by a key informant:
  [translation] “*The drug subsidy formula is a good formula; the mutual and other health insurance options that are developing everywhere. I think this is closer to negotiating a solution with populations and giving them the ability to pay*” (Former policymaker and current head of a public institution).

Conversely, arguments were made in favour of free healthcare, for example, by presenting the disadvantages of partial fee exemptions when it comes to equitable access to care:

[translation] “*You know what’s always the problem with partial subsidies as opposed to free care? The complexity of partial subsidies. They’ve always created equity issues, because the criteria for receiving a partial subsidy, the subsidy rates, the criteria and everything else, have always been very complex*” (Health professional, head of a health NGO).

In addition, unlike the moral conceptions of free healthcare inspired by local traditions, users want and even demand free healthcare delivery to the extent possible, some looking back with nostalgia on the welfare state of the past. In a tailor-made exemption situation, some users would like these exemption options to be harmonised by extending the free healthcare currently applied to preventive malaria care to curative care. However, some adverse effects are blamed on care providers, users and poor functioning of the healthcare system. To begin with, it was reported that the criteria for distributing insecticide-treated mosquito nets to women during prenatal consultations (depending on whether they will use them regularly or on other unofficial prerequisites) differ between structures, which opens the door to abuse by some ill-intentioned care providers. This was also the case for health centres distributing free drug supplies to indigents. The health ministry made this decision in 2005 without formal eligibility criteria; hence, health officers had to improvise and take initiative [48]. Several cases of sale of freely distributed mosquito nets by insensitive users were also reported. The same goes for unwarranted use of health centres by some users to obtain drugs that are then distributed to their friends and relatives or to other people.

These practices, which are far from the humanist spirit of free healthcare that removes the financial barrier to healthcare access, especially for the poor, prove the need for additional reforms that allow the health system to meet target objectives while minimising adverse effects. This implies using a more systemic, non-vertical approach while moving away from certain provisions of the ‘Pasteurian paradigm’ [14].

The results of the three studies agreed that there were differing assessments among care providers that are generally negative toward free healthcare and users who express their adherence to and satisfaction with widespread free healthcare. Additionally, differences were also obvious between the health professionals who reject the principle of free healthcare without corresponding obligations that they find difficult to perpetuate and those that do accept it and suggest introducing criteria for fair access based on social and economic status. In this case, attention is given to the poor, who are supposed to be fully exempt from paying healthcare costs, as opposed to other classes of individuals for whom it is understood that the applicable exemption must be proportional to their income level.

Some specificities arise for malaria interventions as opposed to HIV/AIDS interventions. There are two aspects regarding payment exemptions for preventive and curative malaria care. The first is the stakeholders’ (decision-makers, care providers) preference for subsidies. The second is that several informants mentioned that the vagueness of the measures taken or the lack of clear guidelines for applying them are the cause of the multifaceted dysfunctions during implementation and that are added to the routine dysfunctions of the health system [29]. This shows the problem of including these actions in stabilised public policies that meet operational effectiveness requirements [49] or requirements that are likely to bring about local reforms [50].

For TB and HIV/AIDS, it appears that almost all diseases investigated received free check-ups, TB drugs and hospitalisation, as stated in the reference texts. The situation is different for HIV/AIDS because delays were reported in placing patients on treatment and reductions in the normal assignment of inputs, but especially in paying for drugs to treat opportunistic infections, as well as in paying fees for biological examinations that are supposed to be free. Care providers also reported many drug and reagent shortages and examination equipment breakdowns.

## Discussion

Table 2 shows a comparison among various characteristics of the interventions considered.

The following elements were used for comparison purposes:

1. The nature of the total exemption (free) or partial exemption (subsidy to varying degrees)
2. The pathology (TB, HIV/AIDS, malaria)
3. The target approach (preventive or curative)
4. The target group
5. The existence of official texts for implementation
6. Budget commitment (Government and international partners)
7. How to access information on the intervention and the services provided

What emerges from the chronological and typological analysis is that, despite government efforts to spatially extend health infrastructures [51], some regions are put at a disadvantage in the overall distribution, including the care supply structural difficulties added to the situational risks (irregular funding, input shortages, lack of infrastructure, equipment and staff). Seen from this perspective, the national malaria care subsidy receives better service delivery coverage at all levels of the health system and in the community; this coverage includes home-based malaria treatment. However, delivery of services related to HIV/AIDS and TB is more limited in space (case of authorised care structures). Because of that, there is unequal access to care in areas that have conventional institutions and those that do not have as many of them.

The experiences described show the wide range of possibilities that the Burkina Faso government has to fund one-time exemptions for targeted healthcare services from large subsidies granted by international organisations. The sudden emergence and the asymmetrical wording of the exemptions considered in the studies illustrate the difficulties in implementing an exemption policy that can integrate the variety of services covered and the consideration of the target groups’ needs based on the nature of the intervention. The existence of ongoing funding (case of TB) that has long been ensured by partners and the Burkina Faso government is in sharp contrast with the one-time resources allocated for most of the fee exemptions from the funding handed out by international organisations. Although the permanence issue [52] should be put into perspective by focusing much more attention on the health results, mainly in favour of the poor and of strengthening the health system, a separate line in the budget for specific exemptions is a huge asset. The same applies to the existence of regulatory and/or legislative texts (case of TB for both aspects stated, unlike other interventions) that constitutes a significant difference in the typology of the exemption schemes from our analytical perspective.

Except for TB, malaria and HIV/AIDS, healthcare fee exemption decisions were made suddenly in the wake of significant events. First, WHO’s decision to use a new malaria treatment drug; second, the pressure from NGOs and community organisations and multilateral organisations (including WHO and the World Bank) to move to free healthcare (case of ARV drugs for adults); third, the possibilities of financing from funders, especially the Global Fund. The combination of these factors was decisive for the concurrent arrival of several sectorial fee exemptions. The significance of funds mobilised over a relatively short period is a reminder of past experiments undertaken in the fight against major endemic diseases. However, the issues were different regarding the nature of the interventions and the impact on the functioning of the health system. For major endemic diseases, these interventions consisted of eradication campaigns in areas determined from vertical programs that use human and financial resources not directly integrated into the health system. This is not the case for exemptions undertaken with international recommendations whose formulation and implementation fall under government jurisdiction. These are inserted into the health system and have implementation gaps and problems because of insufficient preparation and often inappropriate material and human resources.

The overall observation is that exemptions are implemented without first assessing the needs of the health structures involved, including staff, infrastructure and the proper equipment to meet the new requirements. The situation is different for TB interventions, which are carried out in specialised structures that have been in place since the fight against major endemic diseases started. This contributes to weakening the capacities of several healthcare institutions that cannot receive immediate government support or funding to strengthen their abilities to meet new technical requirements. Of course, the health ministry provides for infrastructure and equipment, but this is based on a schedule that is out of step with the needs related to specific interventions such as fee exemption measures often taken suddenly and applied quickly despite previously stated constraints.

In some cases (ARV drugs, TB), the exemption covers only part of the costs (ARV drugs for HIV/AIDS but not the costs of biological monitoring) or takes effect only after the diagnosis is confirmed following an expensive process (case of TB). In these cases, the negative effects focus much more on access to services for the poor, who must often pay unforeseen indirect costs that may be catastrophic and are contrary to their expectations and beyond their means [53].

Theoretically, reducing or eliminating healthcare fees should, above all, benefit the poor, who cannot pay health costs. We highlighted two different situations regarding this. The first was that benefits related to preventive and curative malaria treatment in the interventions studied (distribution of insecticide-treated mosquito nets and ACTs in health centres and among community stakeholders) have an extended distribution channel that is fully accessible upon request. However, those related to HIV/AIDS and TB interventions (biological examinations and check-ups, ARV drug distribution, patient monitoring) are focused on specialised or authorised structures (district, regional or general national referral hospitals or ambulatory treatment centres for HIV/AIDS, TB screening and treatment centres). This means that access to benefits is first limited by the geographic availability of the healthcare services, which, in countries like Burkina Faso, shows major disparities between urban and rural areas and is based on the unequal implementation of national health structures.

Regarding the conditions for access to healthcare delivery, there are other constraints for health services users, especially the poor. The purpose of the exemptions is to remove or lessen the financial accessibility barriers. However, it appears that applying the measures cited exposes eligible women and children to the usual dysfunctions of the health system that do not always allow effective access to free healthcare delivery. This means that, ironically, it is not always possible for users to have access to free healthcare for at least four reasons [11]. Free healthcare is far from being total because of the following shortcomings: (1) direct costs not officially covered in the free benefits package; (2) indirect costs (travel, miscellaneous costs not related to care by family and friends); (3) illicit costs (unjustified invoicing, gifts to health officers, illicit sale of drugs, or other favours asked for fraudulently in exchange for access to care); and (4) purchases imposed on users because of shortages of free drugs or other inputs. This relates to the challenges that users face in accessing care in structures that meet the required standards. Therefore, the study on ARV drugs and the associated benefits showed that patients must travel from their often-remote homes to care centres, which requires indirect costs for travel and accommodation during the time required to receive treatment, and costs related to certain paid services and to amenities during the stay (food, accommodation). These expenses may prove catastrophic to the poorest people.

Regarding the funding of fee exemptions that pertain to the interventions studied, experience shows that two key methods have an impact on the functioning of the health system. The total or partial fee exemption, funded mainly through the government budget (case of TB among the studies considered and other interventions such as prenatal and postnatal consultations for women, vaccinations for children up to 5 years of age and for pregnant women, and care of severe malaria), differs from the total or partial fee exemption funded mainly by international organisations with a minor contribution usually requested from the government. The first case involves stable, long-lasting funding (since the colonial period for TB) often relying on official texts (this was the case most recently with the childbirth and emergency obstetric and neonatal care subsidies). This allows long-term follow-up and evaluations to be conducted to assess, among other things, the effects on recipients and on the health system. In the second case, for about 10 years, there have been successive requests for funding from the Global Fund to scale up the fight against HIV/AIDS, TB and malaria. This funding is for a limited time and raises questions about the possibility of continuing the interventions after the funders’ commitment ends.

It is around these actions that the debate between supporters of a total fee exemption and supporters of subsidies becomes clear. Therefore, the concept of a welfare state re-enters the equation and the lack of contribution from recipients is called into question by bringing up lesser ‘responsibility’ and other arguments that sometimes draw their inspiration from cultural concepts. Unlike the adverse effects of addiction to aid [54], recent exemption initiatives exclusively covered by the government budget prove that there are endogenous solutions for funding major interventions in health or in other areas. Indeed, there has been an unprecedented commitment since March 2016 to free health services for mothers and children (free care for pregnant women and for children under five, for caesarean sections, for treatment of obstetric fistulas). In this context, the basic question is no longer about the sustainability of the interventions, but about the government’s ability to keep this level of financial commitment within the health budget (7.29% of the 2015 budget). The political will is there, but several challenges, including introduction of universal health insurance, are difficult to take on for a low-income country that receives significant budgetary support from international aid.

Another aspect is that the poorest are less inclined to turn to healthcare services by themselves, even when care is free. In that respect, action research conducted in rural areas [48] illustrates the effects of social exclusion, which is more extensive than the stigmatisation that victimises the poor. Experience shows that these effects make their presence felt through the low use of care by indigents who receive free care totally covered by peripheral health centre management committees. The same is true for care that benefits from an exemption scheme and immediately helps people who have the will and the means to go into authorised structures; the difference lies in the poor, who are faced with other obstacles more related to individual dispositions, social taboos and geographical accessibility constraints. Indeed, support is necessary to guarantee access to care for this class of vulnerable individuals [55].

We argue that exemptions to paying for care introduce dispositions that favour better access to care for the poor, but this is not possible in the conditions described for vulnerable persons targeted by public policies. In practice, the poor have a guaranteed – and often facilitated –right to access to free care, but exercising this right is subject to the constraints described above. Hence, the opportunity to implement additional provisions (easy access for specific, and especially vulnerable, groups) to make the exemption measures more effective.

## Conclusion

### In addition to fee exemptions, the quest for fairness and effectiveness of public policies

Attention focused on various areas of targeted healthcare fee exemptions shows an interest in such interventions that are supposed to contribute positively to improving access to healthcare for the most vulnerable and to strengthening the health system. Experience shows that the effects are mitigated, which is partly explained by the often-sudden implementation without enough preparation and without official orientation texts or a broad dissemination of information to all classes of the population. As a perfectible response to the target groups’ needs and to the weaknesses in the provision of care, fee exemptions provide an opportunity to strengthen the health system [56, 57]. In principle, targeted exemptions that are part of focused public policies are a catalyst for vulnerable groups to access healthcare. Conversely, unfavourable conditions for implementing such conditions reveal ordinary dysfunctions in the health system [58]. For example, a lack of medico-technical materials for a health centre that receives around 20 people a day could go unnoticed. However, when staff is tripled because of a targeted payment exemption, the impact that may have on the availability and quality of healthcare delivery is understood. The contexts of applying healthcare fee exemptions provide information on the complexity of the problems to be solved by public health policies and by human, financial and material resources, and necessary reforms. In some cases, inconsistencies or discrepancies were observed between predictions and reality in the implementation of the interventions. Therefore, exemptions intended to be total do not always apply in real time (variation from one structure to the next in paying for certain services). The same is true for selective free healthcare when one part of the care package is concerned (case of drugs), unlike other paid services (case of biological examinations for HIV/AIDS and TB).

The evolution of government options in Burkina Faso (reluctance in the face of total fee exemptions on a national scale after the free care of the welfare state and adoption of cost recovery) shows the extent of unpredictability of a public health policy that is not consistent with a permanent guideline. In this case, recent policy changes likely acted as a catalyst in the adoption of a series of total fee exemption measures that were expected for many years. However, the government made unprecedented commitments that required sustained investment for the duration of the interventions concerned. The issues are health-related (meeting heath objectives that warrant government commitment and enhancing the effectiveness of the health system through large-scale implementation), political (successfully implementing interventions that are part of the national health and development policy), and social (having the covered population, especially the poor, benefit from the services included in the official plans). As with other previous major interventions (cases of the national subsidy for childbirth and emergency obstetric and newborn care that end logically with a broader measure for the same target group), the lack of probative data in the implementation process has, to date, been the weak link in the national healthcare fee exemption system. How can resource allocation decisions be justified without basing them on probative data from validated evaluations? It demonstrates more than ever the interest of reviewing the overall national public health policy approach by having a more prospective vision that enables decision-making in real time and guards against quick implementation without sufficient preparation, which was the common ground of the major interventions to date.

## Abbreviations

ACT: artemisinin-based combination therapy
ARV: antiretroviral drugs
CAMEG: Centrale d’Achat des Médicaments Essentiels et Génériques [central warehouse for essential and generic drug purchases]
CHSA: centre for health and social advancement
LLIN: long-lasting insecticide-treated mosquito net
MDGs: Millennium Development Goals
NGO: non-governmental organisation
PCF: primary care facility
PNT: Programme National de lutte contre la Tuberculose [national tuberculosis control program]
TB: tuberculosis.
